# A multiyear time series (2004–2012) of bacterial and archaeal community dynamics in a changing Arctic Ocean

**DOI:** 10.1093/ismeco/ycad004

**Published:** 2024-01-10

**Authors:** Susanne A Kraemer, Arthi Ramachandran, Vera E Onana, William K W Li, David A Walsh

**Affiliations:** Environment and Climate Change Canada, Montreal, Quebec, H2Y 2E7, Canada; Department of Biology, Concordia University, Montreal, Quebec, H4B 1R6, Canada; Department of Biology, Concordia University, Montreal, Quebec, H4B 1R6, Canada; Department of Fisheries and Oceans, Bedford Institute of Oceanography, Dartmouth, Nova Scotia, B2Y 4A2, Canada; Department of Biology, Concordia University, Montreal, Quebec, H4B 1R6, Canada

**Keywords:** ocean, climate change, marine microbiology

## Abstract

Climate change is profoundly impacting the Arctic, leading to a loss of multiyear sea ice and a warmer, fresher upper Arctic Ocean. The response of microbial communities to these climate-mediated changes is largely unknown. Here, we document the interannual variation in bacterial and archaeal communities across a 9-year time series of the Canada Basin that includes two historic sea ice minima (2007 and 2012). We report an overall loss of bacterial and archaeal community richness and significant shifts in community composition. The magnitude and period of most rapid change differed between the stratified water layers. The most pronounced changes in the upper water layers (surface mixed layer and upper Arctic water) occurred earlier in the time series, while changes in the lower layer (Pacific-origin water) occurred later. Shifts in taxonomic composition across time were subtle, but a decrease in *Bacteroidota* taxa and increase in *Thaumarchaeota* and *Euryarchaeota* taxa were the clearest signatures of change. This time series provides a rare glimpse into the potential influence of climate change on Arctic microbial communities; extension to the present day should contribute to deeper insights into the trajectory of Arctic marine ecosystems in response to warming and freshening.

## Main text

The Arctic Ocean is rapidly changing. Surface waters are warming due to climate change and sea ice retreat [1], and freshening due to melting sea ice and river runoff [2, 3]. Warming and freshening are having profound effects on Arctic marine ecosystems [4], including reduced nutrients [5] and shifts in primary production [6]. Although changes in phytoplankton communities have been documented through time series observations [7-9], and a single study has report on temporal dynamics of microbial diversity over the 2007 sea ice minimum [10], there is a conspicuous lack of information on the change in bacterial and archaeal diversity over interannual time frames relevant to climate change.

**Figure 1:**
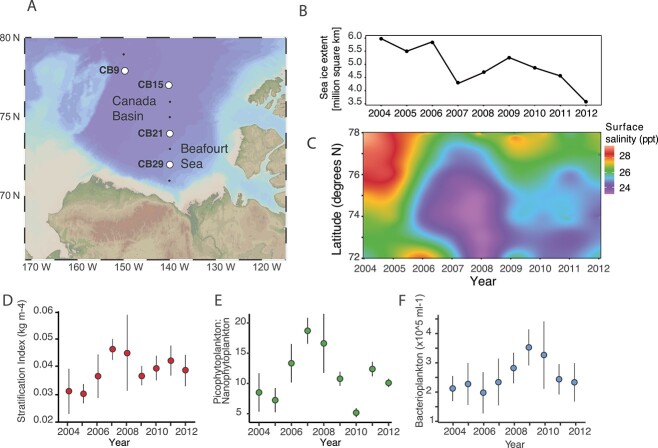
The change in environmental conditions in the Canada Basin over the 2004–2012 time series; (A) map of the Arctic Ocean showing station locations (circles) included in this study; (B) variability in summer sea ice extent in the Arctic Ocean; data are from the National Snow and Ice Data Center; (C) the latitudinal gradient in sea surface salinity over the time series; (D) stratification index, error bars indicate among-station standard deviation; (E) ratio of picophytoplankton to nanophytoplankton; (F) bacterioplankton. In panels E and F, values are the average of all measurements >15 m in depth and salinity <31 PSU, and error bars indicate among depth and among-station standard error.

In this study, we documented the variation in bacterial and archaeal communities in the Arctic Ocean (Canada Basin) across a 9-year time series of summer waters, encompassing two historic sea ice minima (2007 and 2012) (Fig. 1A and B). The dataset encompassed four stations (CB29, CB21, CB15, and CB9) along a latitudinal transect of the Canada Basin (72^o^–78^o^ N) (Supplementary Table S1). Samples were analyzed from three water layers comprising the relatively fresh summer mixed layer (SML; 3–9 m), the upper Arctic water (UAW; 16–78 m) underlying the SML, and the deeper Pacific-origin water (PW; 49–154 m) bounded by 31–33 practical salinity units (PSU). The dataset captured the temporal variation in environmental conditions previously reported from a spatially more extensive time series of the Canada Basin [11]. In particular, surface water freshening and increased stratification associated with sea ice melt were apparent (Fig. 1C and D). Similarly, the previously observed shift toward smaller phytoplankton cell size and a later increase in bacterioplankton abundance were also captured in the dataset (Fig. 1E and F).

**Figure 2:**
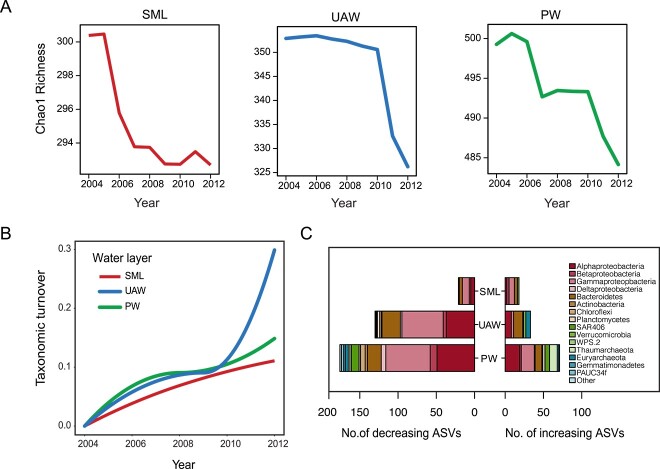
The change in bacterial and archaeal communities in the Canada Basin over the 2004–2012 time series; (A) partial dependency plots of changes in Chao-1 richness predicted across the time series by the random forest approach; (B) the magnitude and rate of change in community composition (taxonomic turnover) across the time series as predicted by generalized dissimilarity modelling; (C) the number and taxonomic composition of bacterial and archaeal ASVs that significantly increased or decreased across the time series.

Bacterial and archaeal diversity was assessed using a universal filter-polymerase chain reaction (PCR) approach [12, 13] targeting the V4 region of the 16S rRNA gene. From 179 samples, we generated 6851 amplicon sequence variants (ASVs) (Supplementary Table S2, Supplementary Fig. S1). Chao-1 estimates of ASV richness were significantly correlated with sampling year, latitude, and depth for the UAW and PW layers, but not for the SML layer (Supplementary Table S3). We modeled the nonlinear influence of oceanographic variables, including the sampling year, on ASV richness using a random forest approach. Random forest models explained 39%–54% of the variation in ASV richness (Supplementary Table S4). Of all variables, year best explained changes in ASV richness in the UAW (27.7%) and was also significant for the SML (13.3%) and PW (4.3%). Visualizing the relationship between year and modeled ASV richness revealed a temporal decline in richness across all water layers (Fig. 2A). Remarkably, the period of most sudden decline varied between water layers. Single declines in ASV richness were observed in 2005–2007 for the SML and in 2010–2012 for the UAW, while a two-step decline was observed in the PW.

We next investigated changes in community composition (i.e. turnover) using generalized dissimilarity models (GDMs). GDMs allowed us to model both the rate and magnitude of change in turnover across the time series and in response to oceanographic variables. Sampling depth and year were the two most important variables in a GDM constructed from the full dataset (Supplementary Table S5). When GDMs were constructed for each of the three water layers separately, sampling depth and year were still significant, but remarkably sampling year was the top predictor of community turnover in the UAW (Supplementary Table S5). The period of most rapid turnover in the UAW was 2010–2012 (Fig. 2B), coinciding with the previously observed decline in richness (Fig. 2A). A similar overlap was observed for the PW, where community turnover mirrored the decline in richness over the time series, but was not significant.

The combined observation of an overall decrease in diversity and corresponding shift in community composition suggested a pattern of taxon loss across the time series. So, we next used Threshold Indicator Taxa Analysis to identify ASVs that significantly changed in each of the three water layers. As expected, we consistently identified more ASVs that decreased over time compared to ASVs that increased (Supplementary Table S6, Fig. 2C). Generally, decreasing ASVs represented a higher taxonomic diversity compared to increasing ASVs, and the majority of decreasing ASVs belonged to *Proteobacteria* (16–152 across water layers) and *Bacteroidetes* (4–24 across water layers). A significant number of increasing ASVs were from *Archaea* (18 across the UAW and PW); *Euryarchaeota* ASVs increased in the UAW (6), while *Thaumarchaeota* ASVs increased in the deeper PW (10).

An earlier study in the Arctic Ocean documented microbial community composition before and after the 2007 sea ice minimum [10]. Our study, which covers a larger oceanographic region and an extended time series, builds on this seminal study. We observed patterns that were consistent and some that were in contrast to the previous work. For example, we observed a loss in *Bacteroidetes* diversity as previously reported. *Bacteroidetes* are involved in the degradation of complex organic matter produced by phytoplankton [14, 15] and changes may indicate a response to shifting phytoplankton communities. In contrast to the previous report of a decrease in ammonia-oxidizing Archaea (AOAs) within the *Thaumarchaeota*, we detected a signal for an increase in AOA diversity. Seasonal changes in Arctic *Thaumarchaeota* linked to shifting water masses have been previously reported [16], and given the importance of AOAs in the nitrogen cycle [17], further in-depth investigations on their temporal dynamics are warranted.

This study provided evidence that significant shifts in bacterial and archaeal community composition occurred between 2004 and 2012, a period that coincided with two historic sea-ice minima. Although changes in community diversity and composition were associated with nutrient concentrations and phytoplankton composition (Supplementary Tables S3 and S4), the links should be interpreted cautiously; significant vertical gradients in these variables were present in single water masses, challenging our ability to disentangle depth versus temporal structure driving these relationships. Instead, we conservatively documented the overall interannual changes in the community, rather than the specific environmental drivers of change. Overall, this relatively short time series provided a rare glimpse into the influence of climate change on Arctic Ocean microbial communities. Given the inherent interannual variability in oceanographic conditions, extension of the time series to the present day and the inclusion of regional variation in ice cover extent, will provide deeper insights into the trajectory of Arctic ecosystems and the consequences of a warmer, fresher Arctic Ocean [18, 19].

## Supplementary Material

Kraemer_etal_Figure_S1_ycad004

Kraemer_etal_TableS1_ycad004

Kraemer_etal_TableS2_ycad004

Kraemer_etal_TableS3_ycad004

Kraemer_etal_TableS4_ycad004

Kraemer_etal_TableS5_ycad004

Kraemer_etal_TableS6_ycad004

Kraemer_etal_SOM_final_ycad004

## Data Availability

The ASV data are included in Supplementary Table S2. The raw data are available at NCBI under accession number PRJNA1040775.
