# Supplementary figures and images for: A multiyear time series (2004–2012) of bacterial and archaeal community dynamics in a changing Arctic Ocean
Source: ISME Commun. 2024 Jan 10;4(1):ycad004. doi: 10.1093/ismeco/ycad004 (PMC10809757; doi:10.1093/ismeco/ycad004)

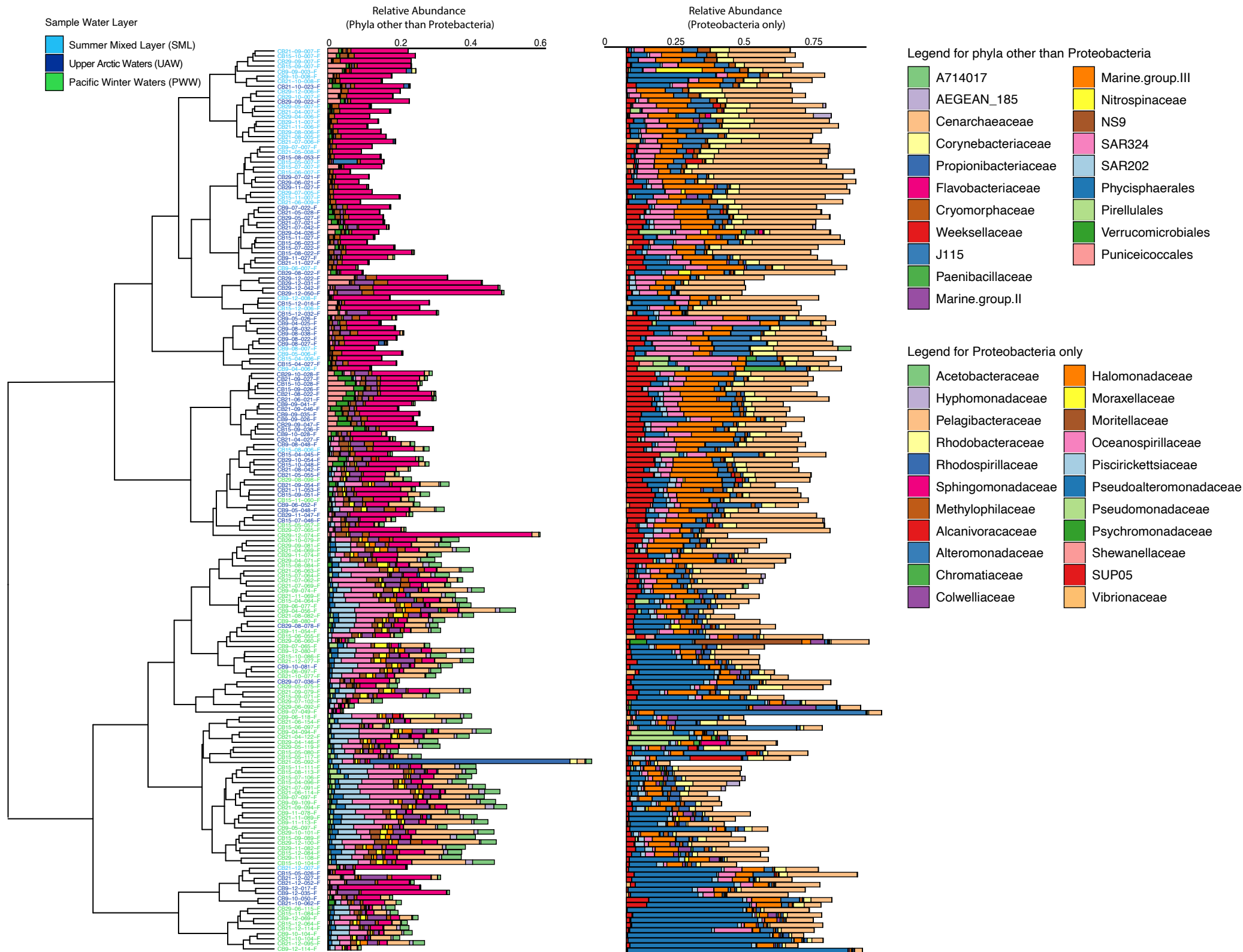

Supplement: Kraemer_etal_Figure_S1_ycad004 [file kraemer_etal_figure_s1_ycad004.pdf]
