# Supplementary material for: A multiyear time series (2004–2012) of bacterial and archaeal community dynamics in a changing Arctic Ocean
Source: ISME Commun. 2024 Jan 10;4(1):ycad004. doi: 10.1093/ismeco/ycad004 (PMC10809757; doi:10.1093/ismeco/ycad004)
Supplement: Kraemer_etal_TableS3_ycad004 [file kraemer_etal_tables3_ycad004.docx]

|  | Year | Latitude | Depth |
| --- | --- | --- | --- |
| SML | 5665 | 6067 | 966 |
| UAW | **46653** | **32967** | **77900** |
| PW | **49398** | **38160** | **64372** |

Table S3. Sums of squares based on anova of Chao1 richness as a function of sampling year, latitude, and depth for each water mass. Bold values indicate significate covariates.
