# Supplementary material for: A multiyear time series (2004–2012) of bacterial and archaeal community dynamics in a changing Arctic Ocean
Source: ISME Commun. 2024 Jan 10;4(1):ycad004. doi: 10.1093/ismeco/ycad004 (PMC10809757; doi:10.1093/ismeco/ycad004)
Supplement: Kraemer_etal_TableS4_ycad004 [file kraemer_etal_tables4_ycad004.docx]

|  | Chao 1 richness | | | |
| --- | --- | --- | --- | --- |
|  | Full | SML | UAW | PW |
| R^2^ fit^1^ | 0.77 | 0.34 | 0.54 | 0.42 |
| OOB^2^ | 0.66 | 0.01 | 0.20 | 0.19 |
| Year | 1.3 | 13.8 | 27.7 | 4.3 |
| Depth | 21.5 | 0.0 | 9.8 | 0.0 |
| Salinity | 11.3 | 20.3 | 3.8 | 7.6 |
| Temperature | 1.1 | 0.0 | 0.18 | 8.6 |
| Nitrate | 14.7 | 0.0 | 14.8 | 14.3 |
| Phosphate | 27.2 | 54.0 | 6.7 | 22.4 |
| Silicate | 12.1 | 0.0 | 7.2 | 19.4 |
| Latitude | 0.7 | 10.6 | 2.4 | 3.0 |
| Longitude | 0.1 | 1.2 | 1.7 | 0 |
| Phytoplankton | 3.3 | 0.0 | 2.2 | 9.9 |
| Nanophytoplankton | 1.9 | 0.0 | 22.8 | 1.2 |
| Picophytoplankton | 3.8 | 0.0 | 0.3 | 8.4 |
| Bacterioplankton | 1.1 | 0.0 | 0.3 | 0.8 |

Table S4. RF results indicating relative importance (as % of explained variation) of the explanatory variables on bacterioplankton diversity.

^1^total amount of variation explained

^2^model error rate in out of bag test
