# Supplementary material for: A multiyear time series (2004–2012) of bacterial and archaeal community dynamics in a changing Arctic Ocean
Source: ISME Commun. 2024 Jan 10;4(1):ycad004. doi: 10.1093/ismeco/ycad004 (PMC10809757; doi:10.1093/ismeco/ycad004)
Supplement: Kraemer_etal_TableS5_ycad004 [file kraemer_etal_tables5_ycad004.docx]

Table S5. GDM results including the model deviance, deviance explained, and predictor importance. Bold values indicate significant predictors.

|  | Full | | SML | | UAW | | PW | |  |
| --- | --- | --- | --- | --- | --- | --- | --- | --- | --- |
| Model deviance | | 607.917 | | 10.014 | | 55.62 | | 113.954 | |
| Percent deviance explained | | 70.413 | | 56.475 | | 55.32 | | 33.053 | |
| Year | | **1.83** | | **4.46** | | **23.35** | | 3.83 | |
| Depth | | **3.59** | | **14.49** | | **12.35** | | 4.4 | |
| Salinity | | 0.022 | | 0.072 | | 0.07 | | 0.13 | |
| Temperature | | 0.052 | | 2.41 | | 1.75 | | 0 | |
| Nitrate | | 0.21 | | 4.13 | | 0.38 | | 0 | |
| Phosphate | | 0.21 | | 4.95 | | 0.67 | | 0 | |
| Silicate | | 0.0045 | | 1.33 | | 1.86 | | 0.45 | |
| Latitude | | **0.57** | | 0.67 | | 1.48 | | **3.42** | |
| Phytoplankton | | 0.017 | | 0 | | 0 | | 0 | |
| Nanophytoplankton | | 0.022 | | 0.066 | | 1.01 | | 0.0.82 | |
| Picophytoplankton | | 0.000002 | | 0.27 | | 0.4 | | 0.28 | |
| Ratio of pico to nano | | **1.17** | | 0.16 | | 0.48 | | 1.76 | |
| Bacterioplankton | | **1.57** | | 4.67 | | 1.07 | | **2.91** | |
