# Supplementary material for: A multiyear time series (2004–2012) of bacterial and archaeal community dynamics in a changing Arctic Ocean
Source: ISME Commun. 2024 Jan 10;4(1):ycad004. doi: 10.1093/ismeco/ycad004 (PMC10809757; doi:10.1093/ismeco/ycad004)
Supplement: Kraemer_etal_TableS6_ycad004 [file kraemer_etal_tables6_ycad004.docx]

Table S6. Summary of threshold indicator analysis with year as the environmental variable.

| Water mass | No. of significantly changing taxa | No. of increasing taxa | No. of decreasing taxa |
| --- | --- | --- | --- |
| SML | 39 | 18 | 21 |
| UAW | 163 | 33 | 130 |
| PW | 314 | 71 | 176 |
