# Supplementary material for: A multiyear time series (2004–2012) of bacterial and archaeal community dynamics in a changing Arctic Ocean
Source: ISME Commun. 2024 Jan 10;4(1):ycad004. doi: 10.1093/ismeco/ycad004 (PMC10809757; doi:10.1093/ismeco/ycad004)
Supplement: Kraemer_etal_SOM_final_ycad004 [file kraemer_etal_som_final_ycad004.docx]

**Materials and Methods (intended for Supplementary Online Materials)**

**Environmental data and sample collection**

Samples were collected between 2004 and 2012 during the Joint Ocean Ice Study (JOIS) in the Canada Basin on the CCGS Louis S. St-Laurent. Sampling was conducted between July and August, except for 2009 and 2010 when sampling was conducted through September to October. The collection and processing of all environmental and oceanographic data used in this study are described in detail in McLaughlin et al. (2012) [1] and Li et al., (2013) [2]. We used the same stratification (kg m^−4^) index as in Li et al. (2013), which was the arithmetic difference of density at 150 m and 0 m, divided by 150 m. Seawater samples (2 mL) used for filter PCR were collected from 5-7 depths across the upper 200 m of the Canada Basin, fixed in 1% paraformaldehyde for 15 minutes at room temperature, flash frozen in liquid nitrogen, and then stored at -80°C for later analysis.

**Filter PCR of 16S rRNA gene amplicons**

For each sample, 500 µl of fixed seawater was filtered through a 25 mm polycarbonate filter with a 0.2 μm pore size (GE) using vacuum filtration (100 kPa). 5 ml of sterilized MilliQ ultra-pure water was passed through the filters three times to remove the residual paraformaldehyde preservative. Filters were sectioned and a sixteenth of each filter was transferred to a 200 ml PCR tube. Direct amplification of the V4 region on the 16S rRNA gene was conducted on filters similar to the approach used in El-Swais *et al.* (2015) [3]. We used the 515F primer (5’-GTG YCA GCM GCC GCG GTA A-3’) from Parada et al. (2016) [4] and the 806R primer 5’-GGA CTA CNV GGG TWT CTA AT-3’) from Apprill et al. 2015 [5]. In combination this primer set enhances the detection of SAR11 and Crenarachaeota/Thaumarchaeota. PCR reactions (50 μL) contained 0.5 μM each primer, 1 X Phire Reaction Buffer, 0.2 mM deoxynucleotide triphosphates and 1 μL (1 unit) of Phire Hot Start II DNA Polymerase (Thermo Scientific). Cycling conditions were as follows: initial 3-minute denaturing step, 30 cycles of 5 seconds at 98°C, 5 seconds at 50°C and 10 seconds at 72°C, and a final elongation step of 1 minute at 72°C. The PCR amplicons, with the CS1 (5’-ACACTGACGACATGGTTCTACA-3’) and CS2 (5’-TACGGTAGCAGAGACTTGGTCT-3’) adapters attached, were purified at Genome Quebec (McGill University) and subsequently sequenced using the Illumina MiSeq 250bp paired-end platform.

**Amplicon data processing**

Raw reads were processed using the DADA2 package in R [6]. Quality profiles were created for all reads. Reads were then trimmed, merged and dereplicated, followed by ASV identification and chimera removal. Before further analysis we removed ASVs found in less than three samples, which likely result from amplification errors. Taxonomy of ASVs was using the GreenGenes 16S rRNA gene database [7]. Further analysis of the ASV table was performed using the phyloseq [8] package in R version 4.2.2. Graphics were created using the ggplot2 package [9].

**Statistical analyses**

Chao1 richness was calculated on a rarefied dataset (18,992 sequences; the size of the smallest sample) using phyloseq [8]. Analysis of variance (anova) of alpha diversity for each water mass was performed using the explanatory variables latitude, year, and depth using R. We used a RF technique from the R party package [10] based on conditional inference regression trees developed by Ryo and Rillig (2017) [11] to model the response of Chao1 richness to environmental variables. 5000 regression trees were used to obtain a stable prediction. A measure of importance was calculated for each predictor variable by cross validating each tree with data not used when the tree was constructed, referred to as the out-of-bag (OOB) data. We generated partial dependency plots (PDPs) illustrating the relationships between Chao1 richness and predictor variable from the RF using the R pdp package [12].

We modeled nonlinear relationships between community turnover (Bray Curtis dissimilarity based on rarified count data) and environmental variables using GDMs as implemented in the gdm R package [13]. Explanatory environmental parameters considered in each model were sampling year, longitude, latitude, bacterial counts per mL, phytoplankton counts per mL, nanophytoplankton counts per mL, picophytoplankton counts per mL, temperature, salinity, nitrate, silicate, and phosphate concentrations, as well as the ration of pico- to nanophytoplankton. GDMs were constructed for the full dataset, as well as for subsets corresponding to each water mass. Significant variables were backwards selected with 100 permutations per step. We conducted taxon indicator threshold analysis for each water mass as implemented in the TITAN2 R package [14] to determine thresholds of turnover over a temporal based on relative ASV abundances. For each water mass, highly prevalent (present in all samples of the water mass) ASVs were removed before analysis.

**References**

1. McLaughlin, F., et al. Physical, chemical and zooplankton data from the Canada Basin and Canadian Arctic Archipelago, July 20 to September 14, 2006, *Can. Data Rep. Hydrogr. Ocean Sci*., 2012; **186**, x + 373.

2. Li WKW, Carmack EC, McLaughlin F a., Nelson RJ, Williams WJ. Space-for-time substitution in predicting the state of picoplankton and nanoplankton in a changing Arctic Ocean. *J Geophys Res Oceans* 2013; **118**: 5750–5759.

3. El-Swais H, Dunn K a., Bielawski JP, Li WKW, Walsh DA. Seasonal assemblages and short-lived blooms in coastal north-west Atlantic Ocean bacterioplankton. *Environ Microbiol* 2015; **17**: 3642–3661.

4. Parada AE, Needham DM, Fuhrman JA. Every base matters: Assessing small subunit rRNA primers for marine microbiomes with mock communities, time series and global field samples. *Environ Microbiol* 2016; **18**: 1403–1414.

5. Apprill A, McNally S, Parsons R, Weber L. Minor revision to V4 region SSU rRNA 806R gene primer greatly increases detection of SAR11 bacterioplankton. Aquat Microb Ecol 2015; 75: 129-137.

6. Callahan BJ, McMurdie PJ, Rosen MJ, Han AW, Johnson AJA, Holmes SP. DADA2: High-resolution sample inference from Illumina amplicon data. *Nat Methods* 2016.

7. DeSantis TZ, Hugenholtz P, Larsen N, Rojas M, Brodie EL, Keller K, et al. Greengenes, a chimera-checked 16S rRNA gene database and workbench compatible with ARB. *Appl Environ Microbiol* 2006; **72**: 5069–5072.

8. McMurdie PJ, Holmes S. Phyloseq: An R Package for Reproducible Interactive Analysis and Graphics of Microbiome Census Data. *PLoS One* 2013.

9. Wickham H. Package `ggplot2`: Elegant Graphics for Data Analysis. *Springer-Verlag New York* 2016.

10. Hothorn T, Hornik K, Zeileis A. Unbiased Recursive Partitioning: A Conditional Inference Framework. *Journal of Computational and Graphical Statistics* 2006; **15**: 651–674.

11. Ryo M, Rillig MC. Statistically reinforced machine learning for nonlinear patterns and variable interactions. *Ecosphere* 2017; **8**: e01976.

12. Greenwell, Brandon M. pdp: An R Package for Constructing Partial Dependence Plots. *R J* 2017; **9**: 421.

13. Fitzpatrick MC, Mokany K, Manion G, Lisk M, Ferrier S, Nieto-Lugilde D. gdm: Generalized Dissimilarity Modeling. *R package version 1422*. 2021.

14. Baker M, King R, Kahle D. An Introduction to TITAN2. 2019; **10**: 1–26.

**Supplementary figure and table titles**

**Figure S1:** Taxonomic composition of bacterial and archaeal communities from the Canada Basin, Arctic Ocean over 2004-2012. The dendrogram was generated by hierarchical clustering of samples based on relative abundance of ASVs using the hclust function in stats package for R with a Bray-Curtis dissimilarity metric). The relative abundance of taxonomic groups represented by greater than or equal to 3.5 % of the 16S rRNA sequences were included in the column charts.

**Table S1.** Sample information and environmental data. Values in red were missing from the original dataset and were interpolated as average values from the same depths or water masses.

**Table S2**. Taxonomic identity and distribution of ASVs from the Canada Basin, Arctic Ocean

**Table S3.** Sum of squares based on anova of Chao1 richness as a function of sampling year, latitude, and depth for each water mass. Bold values indicate significate covariates.

**Table S4.** RF results indicating relative importance (as % of explained variation) of the explanatory variables on bacterioplankton diversity.

**Table S5.** GDM results including the model deviance, deviance explained, and predictor importance. Bold values indicate significant predictors.

**Table S6.** Summary of threshold indicator analysis with year as the environmental gradient variable.
